# Thirty-year trends in clinicopathologic characteristics and prognosis after gastrectomy for gastric cancer: A single institution in Northern China

**DOI:** 10.7150/jca.36927

**Published:** 2020-01-01

**Authors:** Yu-en Tan, Peng-liang Wang, Song-cheng Yin, Chao Zhang, Wen-bin Hou, Hui-mian Xu

**Affiliations:** Department of Surgical Oncology, First Affiliated Hospital of China Medical University, Shenyang, China.

**Keywords:** gastric cancer, time tendency, clinicopathologic characteristics, prognosis.

## Abstract

**Background:** We integrated changes in the trends in clinicopathologic characteristics and postoperative prognosis in patients with gastric cancer Northern China over a 30-year period.

**Methods:** A retrospective analysis of patients undergoing gastric cancer resection and complete follow-up information from January 1981 to December 2010 in the first affiliated Hospital of China Medical University was carried out. We divided the patients into three consecutive periods.

**Results:** A total of 3,520 patients were included in this study. The proportion of lower tumors increased (from 58.8 to 66.9%), while that of upper tumors decreased (from 21.3 to 13.4%). The proportion of tumors > 5cm decreased (from 58.6 to 41.1 %), but the increasing trend of poorly differentiated gastric cancer was obvious (from 60.1 to 75.7%). The percentage of early gastric cancer increased from 10.0 to 15.5 during the study periods, and that of TNM stage Ⅳ cancer decreased from 38.6 to 28.1. In surgery treatment, the rate of radical resection increased to 92.1% in recent period, and the average number of retrieved lymph nodes increased. The 5-year survival rate gradually increased from 36.5% to 48.5% (p<0.001). The Multivariate analysis showed that age, tumor size, T stage, N stage, number of retrieved lymph nodes and resection type were independent prognostic factors for gastric cancer.

**Conclusion:** The patterns of clinicopathologic features for gastric cancer changed during the 30-year period in North China. Overall survival (OS) could be increased by early detection of tumors and standard surgical treatment.

## Introduction

Gastric cancer is the fifth most commonly diagnosed cancer and the third leading cause of cancer death 1. China has a high-incidence of gastric cancer, with about 400,000 new cases each year, and about 350,000 cases of gastric cancer, accounting for 40% of case globally 2, 3. At present, surgical resection is the most effective treatment for gastric cancer, but when distant metastasis occurs, chemotherapy is required.

There are many factors that affect the survival rate of patients with gastric cancer, including tumor grade, and histologic type. Large sample data reveal in detail the impact of clinicopathological factors on the prognosis of patients with gastric cancer 4, 5. Although controversial, it is of great significance to evaluate the efficacy of surgical treatment and carry out prospective clinical research to improve the comprehensive treatment of gastric cancer. In recent years, foreign researchers has become increasingly aware of the importance of changing treads in the clinicopathological factors for gastric cancer 6-9, but there are few similar reports in China 10.

Based on the collected data of gastric cancer patients in China, we assessed the time changes of the clinicopathologic features and postoperative outcome from 1981 to 2010, with the purpose of introducing the present situation and changes of gastric cancer in China.

## Materials and methods

### Patient Source

We obtained retrospective date for patients with gastric cancer treated surgically from January 1981 to December 2010 at the single center of the first affiliated Hospital of China Medical University. We included patients with pathologically diagnosed gastric adenocarcinoma with detailed medical records and complete pathological and follow-up data after surgery. Exclusion criteria were: 1 patients with neoadjuvant therapy; 2 patients who underwent non-tumor resection such as laparotomy and gastrojejunostomy; 3 patients with other gastric malignant tumors (such as gastric lymphoma and gastrointestinal stromal tumor) or postoperative recurrence of gastric cancer; 4 patients with other, simultaneous primary malignant tumors, such as colorectal, or breast cancer; and 5 patients who died from surgical complications during the perioperative period. Eventually, a total of 3520 gastric cancer patients were enrolled in our study.

We divided the patients into three chronological groups in order: 1981-1990 (period 1), 1991-2000 (period 2), and 2000-2010 (period 3). The detailed demographic and clinicopathological features of patients in the three groups are presented in **Table 1**. All the biopsies were performed by pathologists using standardized protocols.

The tumor sites in the stomach were uniformly classified as the upper, middle and lower. The histological types were well/moderately and poorly differentiated carcinoma. In accordance with the eighth edition of the TNM staging system of the American Joint Committee on Cancer/Union for International Cancer Control (AJCC/UICC) 11, the patients were divided into eight subgroups on the basis of their post-operative pathology.

All patients received standardized follow-up, based on telephone interviews or brief messages after discharge, every 3 months in the first 3 years, every 6 months in the fourth and fifth years, and every year thereafter until death or final follow-up (June 30, 2015).

### Statistical analyses

We used SPSS version 20.0 for statistical analysis (SPSS Inc., Chicago, IL, USA). Differences in categorical variables were analyzed χ 2 test. Kaplan-Meier method was used to analyze the overall cumulative survival rate. Differences among the groups were depended on log rank test; A Cox regression model was used for multivariate analysis. Two-sided P < 0.05 was regarded as statistically significant.

## Results

### Demographic characteristics

**Figure 1:**
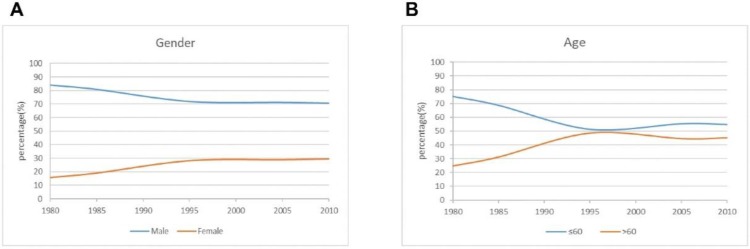
The curve of demographic characteristics changes over time for the patients.

A total of 3520 gastric cancer patients who met the above criteria were included in the study. The number of patients undergoing surgery increased from 581 in the period 1 to 2167 in the period 3. There were 2566 men and 954 women, with a male to female ratio of 2.7: 1, and the proportion of male patients was lower than that in recent years. The mean age was 58.2 years, and the proportion of patients older than 60 years was greater in periods 2 and 3 than in period 1 **(Figure 1).**

### Pathological characteristics

**Figure 2:**
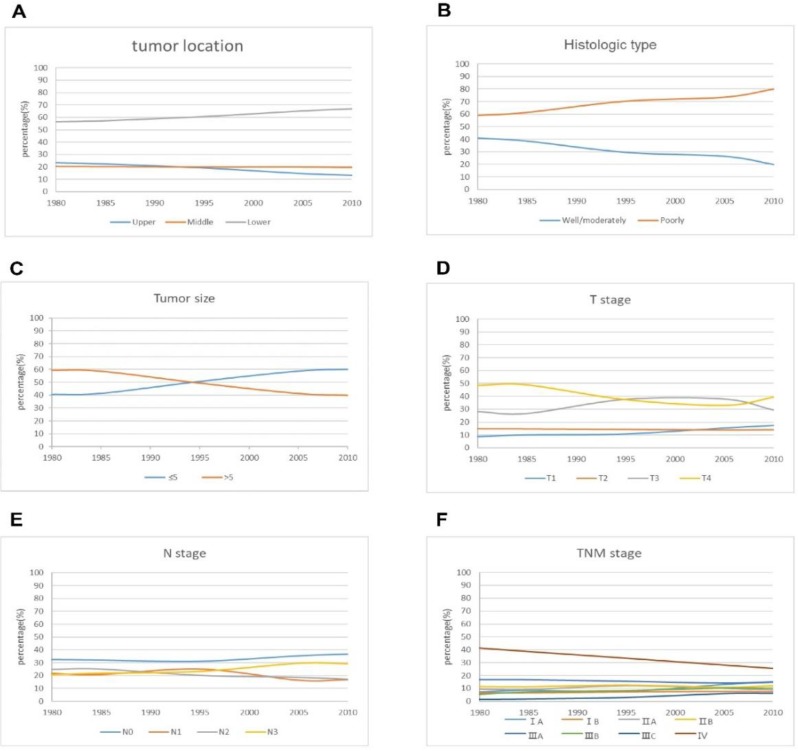
The curve of pathological characteristics changes over time for the patients.

Most tumors were located in the lower stomach. The largest proportion of lower tumors gradually increased from 58.8 % in period 1 to 66.9% in period 3. On the contrary, upper tumors showed a slight downward trend. Changes in the histologic types of tumor in the three periods are shown in **Figure 2**. The proportion of well/moderately differentiated tumors decreased from 39.9 % in period 1 to 24.3 % in period 3, and poorly differentiated tumors showed an upward tendency in the three periods. There was a gradual increase over time in tumors > 5 cm compared with tumors < 5cm. The proportion of patients with early gastric cancer (EGC) increased from 10 % in period 1 to 15.5 % in period 3. There was no obvious trend in relation to N stage. Similar to T stage, the proportion of patients with stage IA curve of TNM stage showed an upward trend, reaching 13.2 % in 3 periods. The proportion of patients with stage IV cancer decreased from 38.6 % in period 1 to 28.1 % in period 3.

### Surgical treatment

**Figure 3:**
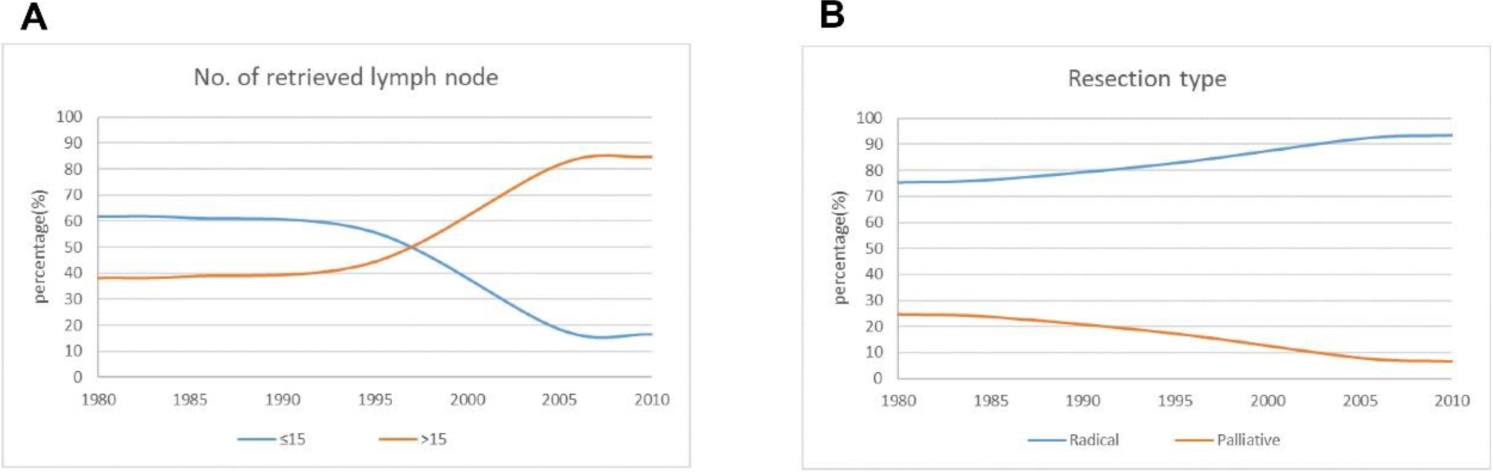
The curve of operative treatment changes over time for the patients.

The number of lymph node metastases is one of the important indexes of clinical stage, and it can be used to evaluate surgical quality. The greater the number of lymph nodes dissected, the more favorable it is to analyze and evaluate the prognosis of patients. In this study, 15 lymph nodes were used as the breakpoint. The proportion of patients with dissection of >15 lymph nodes increased significantly from 38.6 % in period 1 to 81.7 % in period 3. The percentage of patients who underwent radical resection increased significantly, reaching 92.1 % in period 3 **(Figure 3)**.

### Prognosis

**Figure 4:**
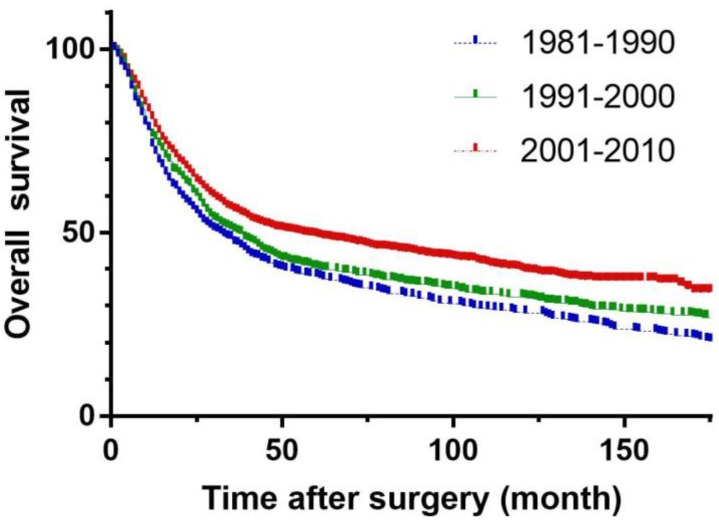
Overall survival of three periods in gastric carcinoma.

To remove the effect of surgical mortality, patients who died 30 days after surgery were excluded. Median follow-up was 138.8 (54.7-431.7) months. The 5-year survival rates in periods 1 and 2 were 36.5 % and 37.8 % respectively, and increased significantly to 48.5 % in the period 3 (p<0.001)** (Figure 4)**.

### Univariate and multivariate analyses

To establish the relationship between clinicopathological factors and prognosis of gastric cancer, we used the Cox regression model to analyze the patients. Univariate analysis showed that factors affecting prognosis of gastric cancer included age, tumor size, T stage, N stage, M stage, number of retrieved lymph nodes, surgical methods and time period. Sex, tumor location, histological type and other factors had no correlation with prognosis of gastric cancer. Multivariate analysis showed that age, tumor size, T-stage, N stage, number of retrieved lymph nodes and resection type were independent prognostic factors for gastric cancer **(Table 2)**.

## Discussion

In recent years, with the development of basic research, the application of radiotherapy, chemotherapy and targeted therapy, medical personnel have a greater understanding of the diagnosis and treatment of gastric cancer and have a direct or indirect effect on the effect of surgical treatment. We analyzed that the time tendency in clinicopathologic characteristics and prognosis after gastrectomy for gastric cancer over a 30-year period in our department of surgery, which is a high-volume center for gastric cancer in Northern China.

Although more young patients with gastric cancer have been reported in recent years 12, 13, gastric cancer still occurs predominantly in middle-aged and elderly patients. Nevertheless, the effect of age on gastric cancer remains controversial. Nakamura et al. 14 has suggested that, for EGC, youth is a protective prognostic factor. However, for advanced gastric cancer patients, young patients are prone to tumor spread and metastasis because of poor tumor differentiation, so prognosis is worse. In contrast, Saito el al. 15 debated that elderly patients undergo limited lymph node dissection and show worse prognosis, which is consistent with the results of our study.

The incidence of gastric cardia carcinoma has gradually improved in western countries 16. However, the proportion of upper gastric tumors is still small, up to 12.3 % in recent time. Our analysis showed that the tumor location wasn't significant in univariate analysis, which is inconsistent with some previous studies 17, 18. The latter suggested that cardia cancer was an independent prognostic factor, because it's frequently undifferentiated or poorly differentiated.

Park el al 19 reported that histologic types were not significantly associated with survival in patients with stage I-III in contrast to stage IV cancer. In our study, there was an increasing trend for poorly differentiated tumor, and the proportion of well/moderately differentiated tumor gradually decreased, which is similar to the research of Wang et al 10. However, our date showed that the significance of histologic types was not obvious in single factor analysis, and was not an independent prognostic factor.

In accordance with previous authors, we conclude that the trend is for an increased incidence in small tumors compared with large tumors 6, 10. Adachi and Won 20, 21 reported that tumor size as an independent factor was connected with the prognosis of gastric cancer, which is in line with our results. Zu el al 22 stated that large tumors were more prone to metastasize than small tumors were, which might explain why large tumors spread more easily via direct invasion or the lymphatics.

As we all know, EGC has better prognosis compared with advanced gastric cancer 23, 24. As a result of popularization of gastroscopy, the proportion of gastric cancer in Japan is almost 40%-60%, which is far higher than in other countries 20, 21. We found that the incidence of EGC gradually increased in recent years despite removing the patients undergoing endoscopic resection. With regard to TNM stage, the general viewpoint is that the prevalence of locally advanced and metastatic tumors, especially stage IV, has decreased in contrast to the prevalence of EGC 7, 8. TNM stage of gastric cancer is an important basis for the selection of clinical treatment strategy and the prognostic judgement in clinical practice. Although the proportion of patients with advanced gastric cancer is decreasing, the total number of patients is still large, and they need more systematic and effective treatment to achieve better prognosis.

The eighth edition of the AJCC UICC/TNM staging system once again emphasizes the criteria of dissection for at least 16 lymph nodes, but for more accurate evaluation, the number of lymph nodes retrieved should be >30. Okajima et al 25 reported that the number of retrieved lymph nodes had a significant effect on survival of patients with stage II and III. In our study, the number of retrieved lymph nodes and resection type were both independent prognostic factors. Radical resection, as a surgical therapeutic tool, is the most effective way to improve the long-term survival rate of gastric cancer. However, many patients with gastric cancer are in the advanced stage during treatment. In the past few years, the deepened understandings of gastric cancer and progress in surgical techniques have increased the proportion of patients undergoing radical resection, especially those with stage Ⅱ or Ⅲ cancer.

The 5-year survival rate in period 3 was better than in periods 1 and 2 in our study, and the time period was statistically significant in univariate analysis but not an independent prognostic factor. These improvements may be correlated with new concepts of gastric cancer treatment, such as canonical, reasonable scope of radical cure, enhancement of comprehensive treatment and individualized treatment, provide new treatment options for patients with gastric cancer. Another new option is the use of neoadjuvant therapy, and a growing number of large clinical trials have demonstrated that radiotherapy, chemotherapy and targeted drugs can help reduce tumor recurrence and improve long-term survival rate 26-28.

The prevalence of clinicopathologic factors, such as radical and palliative resection, has transformed during the 30 years in our department. Overall survival of patients with gastric cancer has significantly improved because of earlier diagnosis and standardized surgical treatment. It is widely believed that gastric cancer has multiple causes, and its prognosis is influenced by many factors, including the environment. Early detection, diagnosis, and treatment are the key to prolonging survival of gastric cancer patients. More people are being added to the early cancer screening program to initiate treatment as early as possible and improve survival time and quality of life.

## Figures and Tables

**Table 1:** Comparison of clinicopathologic features in 3,520 patients with gastric adenocarcinoma after gastrectomy from 1981 to 2010.

Factor	Period 1 n=572, n (%)	Period 2 n=781, n (%)	Period 3 n=2167, n (%)	P valve
Gender				<0.001
Male	463 (80.9)	561 (71.8)	1542 (71.1)	
Female	109 (19.1)	220 (28.2)	625 (28.9)	
Age				<0.001
≤60	393 (68.7)	401 (51.3)	1198 (55.3)	
>60	179 (31.3)	380 (48.7)	969 (44.7)	
Tumor location				<0.001
Upper	122 (21.3)	139 (17.8)	290 (13.4)	
Middle	114 (19.9)	156 (20.0)	438 (19.7)	
Lower	336 (58.8)	486 (62.2)	1449 (66.9)	
Histologic type				<0.001
Well/moderately	228 (39.9)	217 (27.8)	526 (24.3)	
Poorly	344 (60.1)	564 (72.2)	1641 (75.7)	
Tumor size				<0.001
≤5cm	237 (41.4)	395 (50.6)	1270 (58.6)	
>5cm	335 (58.6)	386 (49.4)	897 (41.4)	
T stage				<0.001
T1	57 (10.0)	84 (10.8)	336 (15.5)	
T2	84 (14.7)	112 (14.3)	298 (13.8)	
T3	152 (26.6)	293 (37.5)	818 (37.7)	
T4	279 (48.8)	292 (37.4)	715 (33.0)	
N stage				<0.001
N0	184 (32.2)	244 (31.3)	767 (35.4)	
N1	119 (20.8)	196 (25.1)	353 (16.3)	
N2	143 (25.0)	158 (20.2)	406 (18.7)	
N3	126 (22.0)	183 (23.4)	641 (29.6)	
TNM stage				<0.001
ⅠA	49 (8.6)	61 (7.8)	285 (13.2)	
ⅠB	38 (6.6)	56 (7.2)	162 (7.5)	
ⅡA	53 (9.3)	95 (12.2)	220 (10.2)	
ⅡB	64 (11.2)	98 (12.5)	235 (10.8)	
ⅢA	95 (16.7)	121 (15.5)	305 (14.1)	
ⅢB	41 (7.2)	65 (8.3)	218 (10.1)	
ⅢC	11 (1.9)	24 (3.1)	134 (6.2)	
Ⅳ	221 (38.6)	261 (33.4)	608 (28.1)	
NO. of retrieved lymph node				<0.001
≤15	351 (61.4)	436 (55.8)	396 (18.3)	
>15	221 (38.6)	345 (44.2)	1771 (81.7)	
Resection type				<0.001
Radical	437 (76.4)	647 (82.8)	1996 (92.1)	
Palliative	135 (23.6)	134 (17.2)	171 (7.9)	

**Table 2:** Univariate and multivariate analysis of risk factors in 3520 patients with gastric cancer.

	Univariate analysis				Multivariate analysis			
Variable	SE	HR	95% CI	P valve	SE	HR	95% CI	P valve
Gender	0.046	0.990	0.905-1.084	.834	—	—	—	—
Age	0.041	1.368	1.262-1.482	<.001	0.041	1.318	1.215-1.429	<.001
Tumor location	0.027	0.988	0.936-1.042	.649	—	—	—	—
Histologic type	0.033	0.951	0.891-1.015	.133	—	—	—	—
Tumor size	0.041	1.974	1.822-2.139	<.001	<.001	1.100	1.008-1.200	<.032
T stage	0.022	1.694	1.621-1.771	<.001	0.033	1.193	1.118-1.273	<.001
N stage	0.018	1.625	1.569-1.682	<.001	0.027	1.365	1.294-1.440	<.001
M stage	0.042	2.316	2.132-2.516	<.001	0.100	0.855	0.703-1.040	0.117
No. of retrieved lymph node	0.042	0.766	0.705-0.831	<.001	0.048	0.713	0.648-0.783	<.001
Resection type	0.055	4.022	3.612-4.479	<.001	0.061	2.251	1.997-2.538	<0.01
Time period	0.026	0.831	0.789-0.874	<.001	0.030	0.969	0.914-1.026	0.281
